# Supplementary material for: Griffith theory of physical fractures, statistical procedures and entropy production: Rosetta stone’s legacy
Source: PLoS One. 2023 Nov 3;18(11):e0292486. doi: 10.1371/journal.pone.0292486 (PMC10624320; doi:10.1371/journal.pone.0292486)
Supplement: Attachment — Submitted filename: Response_to_Editor_and_Reviewers.pdf [file pone.0292486.s002.pdf]

**Response to Academic Editor**

Journal Requirements:

When submitting your revision, we need you to address these additional requirements.

1. Please ensure that your manuscript meets PLOS ONE's style requirements, including those for file naming. The PLOS ONE style templates can be found at

https://journals.plos.org/plosone/s/file?id=wjVg/PLOSOne_formatting_sample_main_body.pdf

and

https://journals.plos.org/plosone/s/file?id=ba62/PLOSOne_formatting_sample_title_authors_affiliations.pdf

**Response: We formatted the article according to PLOS ONE's style requirements.**

2. Please note that PLOS ONE has specific guidelines on code sharing for submissions in which author-generated code underpins the findings in the manuscript. In these cases, all author-generated code must be made available without restrictions upon publication of the work. Please review our guidelines at https://journals.plos.org/plosone/s/materials-and-software-sharing#loc-sharing-code and ensure that your code is shared in a way that follows best practice and facilitates reproducibility and reuse.

**Response: The generated code (written in Mathematica) is available in the following repository https://doi.org/10.5281/zenodo.8317810 .**

3. Thank you for stating the following financial disclosure: "No."
At this time, please address the following queries:

    a) Please clarify the sources of funding (financial or material support) for your study. List the grants or organizations that supported your study, including funding received from your institution.
    b) State what role the funders took in the study. If the funders had no role in your study, please state: "The funders had no role in study design, data collection and analysis, decision to publish, or preparation of the manuscript."
    c) If any authors received a salary from any of your funders, please state which authors and which funders.
    d) If you did not receive any funding for this study, please state: "The authors received no specific funding for this work."

Please include your amended statements within your cover letter; we will change the online submission form on your behalf.

**Response: The authors received no specific funding for this work.**

4. Thank you for stating the following in your Competing Interests section: "NO authors have competing interests"
   Please complete your Competing Interests on the online submission form to state any Competing Interests. If you have no competing interests, please state "The authors have declared that no competing interests exist.", as detailed online in our guide for authors at http://journals.plos.org/plosone/s/submit-now

   This information should be included in your cover letter; we will change the online submission form on your behalf.

**Response: The authors have declared that no competing interests exist.**

5. In your Data Availability statement, you have not specified where the minimal data set underlying the results described in your manuscript can be found. PLOS defines a study's minimal data set as the underlying data used to reach the conclusions drawn in the manuscript and any additional data required to replicate the reported study findings in their entirety. All PLOS journals require that the minimal data set be made fully available. For more information about our data policy, please see http://journals.plos.org/plosone/s/data-availability.

   Upon re-submitting your revised manuscript, please upload your study's minimal underlying data set as either Supporting Information files or to a stable, public repository and include the relevant URLs, DOIs, or accession numbers within your revised cover letter. For a list of acceptable repositories, please see http://journals.plos.org/plosone/s/data-availability#loc-recommended-repositories . Any potentially identifying patient information must be fully anonymized.

   Important: If there are ethical or legal restrictions to sharing your data publicly, please explain these restrictions in detail. Please see our guidelines for more information on what we consider unacceptable restrictions to publicly sharing data: http://journals.plos.org/plosone/s/data-availability#loc-unacceptable-data-access-restrictions . Note that it is not acceptable for the authors to be the sole named individuals responsible for ensuring data access.

We will update your Data Availability statement to reflect the information you provide in your cover letter.

**Response: The minimal underlying dataset is generated by the code that outputs a txt file (S1_Dataset.txt). An example of the txt file was uploaded as supporting information to PLOS ONE.**

6. We note that you have stated that you will provide repository information for your data at acceptance. Should your manuscript be accepted for publication, we will hold it until you provide the relevant accession numbers or DOIs necessary to access your data. If you wish to make changes to your Data Availability statement, please describe these changes in your cover letter and we will update your Data Availability statement to reflect the information you provide.

**Response: The provided code (https://doi.org/10.5281/zenodo.8317810) outputs the minimal underlying data set as a txt file. The txt file (S1_Dataset.txt) was uploaded as supporting information to PLOS ONE.**

7. Please review your reference list to ensure that it is complete and correct. If you have cited papers that have been retracted, please include the rationale for doing so in the manuscript text, or remove these references and replace them with relevant current references. Any changes to the reference list should be mentioned in the rebuttal letter that accompanies your revised manuscript. If you need to cite a retracted article, indicate the article's retracted status in the References list and also include a citation and full reference for the retraction notice.

**Response: We have reviewed our reference list, and it is complete and correct.**

**Response to Reviewers**

Dear Editor,

Thank you for giving us the opportunity to submit a revised version of our manuscript titled "Griffith theory of physical fractures, statistical procedures, and entropy production: Rosetta stone's legacy" to Plos One. We carefully considered the reviewer's comments and tried our best to address every one of them. The changes are highlighted within the manuscript.

If you have any concerns or need other requirements, please feel free to contact us.

In the following, we provide a point-by-point response to the comments and concerns.

Kind regards,

**Reviewer's comments to the authors**

**Comment 1:** *The introduction part introduces too much about the stele itself and too little about the experimental process and research direction of this paper. It is suggested to adjust the proportion of the introduction part to highlight the theme.*

**Response:** Thank you for pointing this out. We added a new paragraph that explains more about the experimental process and shrunk the paragraphs related to the stele.

> "In this work, we propose a model based on the use of random numbers (such as Monte Carlo) [5] to describe the fracture process of a solid material in terms of its number of vertices. Roughly speaking, we model the solid as a square polygon and propose that there are breakable points, called" breaking points", on each side of the square. A fracturing event or vertex loss starts at a random breaking point on one side of the square and propagates to another random breaking point on another adjacent side. This fracturing occurs at each vertex, reaching a final state with a few vertices ranging between 4 and 8. Next, M copies of this system (statistical ensemble) are created, which evolve individually in the same way as aforementioned. By counting the area and the number of vertices of each copy at its final state, we derive a probability distribution and use it to find the most likely polygon. As an application of the model, we use this distribution and the Griffith criterion to study the fracture process of the Rosetta stone and conjecture about its eventual future."

**Comment 2:** *The design part of the model uses a square model with a side length of 2cm, while the actual stone tablet was a rectangle before it was broken. It is suggested to add a contrastive description of square and rectangle in the model design to improve the rigor of the experiment.*

**Response:** We added a paragraph that contrasts the probability distribution of a square and a rectangle.

"In this sense, the numerical experiment was carried out using a square and another rectangular shape as initial polygons. The two had six breaking points, evenly distributed on each of their edges. Our results show that the probability distribution for the number of vertices is the same in both cases, namely, there is no difference if we use a square or a rectangle. Evidently, this happens only when we focus solely on its number of vertices. Here, for the sake of simplicity, we show only the results for the square with a side length unity"

**Comment 3:** *In the design of the model, the numerical result directly illustrates the same loss area as the square with 6 breakpoints. It is suggested to add 5 or other breakpoints corresponding to the data and stone tablet actual data comparison process.*

**Response:** Thanks for the suggestion. Certainly, the actual simulation is set to six breaking points, whereas our illustrative Figures 2 and 3 have only five breaking points. We have changed the respective figures and set them to six breaking points.

**Comment 4:** *2 Take the Rosetta Stone as an example. Part of the assumption is that the surface of the stone changes from 4 vertices to 6 vertices.*

**Response:** Thanks for the comment. We have added your suggestion to our article (Section 5, paragraph 2).

"Part of the assumption is that the surface of the stone changes from 4 vertices to 6 vertices."

**Comment 5:** *It is suggested that the definition of entropy should be added before the formula of entropy, so as to facilitate readers' understanding of this part.*

**Response:** Thank you for this suggestion. Accordingly, we added the definition of entropy before equation 3.

"This concept is an essential part of thermodynamics, statistical mechanics [Landau-Toda], and information theory [Volkestein]. For a physical system at a given energy, the number of states accessible to the system (degeneracy) is related to the volume V occupied by the system in phase space. Important, for our purposes, the phase space includes the physical space. Entropy in general terms is defined by the standard relation $S \propto \ln(V)$ due to Boltzmann." (corregir articulo)

**Comment 6:** *The conclusion is suggested to be rewritten. The conclusion of this paper is the description and summary of the previous experimental results, without innovation and sublimation part of the description.*

**Response:** We thank the referee for this comment. Effectively revised the conclusions, we have rewritten the terms indicated (emphasizing innovation, important and novel aspects

developed in our work). The conclusions have also been written promoting more generic aspects.

> "VI. Conclusions. In this work, we have developed a numerical-statistical model to predict the probability of ruptures in a laminar-shape solid (Figures \ref{sq} and \ref{Rosette}). Starting with an initial geometry, we find the most likely configurations in the systematic process of ruptures. While it is true that we consider an initial geometry of a square, this methodology can be adapted to other parallelepipeds.
>
> Given the conditions imposed in our model with four initial vertices, in the generic process of ruptures, the probabilities of reaching six and seven vertices were explicitly calculated (probabilities of 0.35 and 0.34 respectively). Currently, being our main application, the Rosetta Stone has six vertices (three fractures). Then, according to our calculations, there is an acceptable probability of a new fracture (seven vertices) in this archaeological artifact. Future applications to other stelae or archaeological objects will be considered.
>
> Using Griffith's criterion, for solids under stress, it was possible to infer the sequential history of the three Rosetta Stone fractures, even conjecturing the relative size of an eventual fourth rupture.
>
> On the other hand, in this work, we have considered the entropy of the system from the point of view of missing information (fragment dispersion). This exhibits formal mathematical similarity to the Joule expansion in thermodynamics. In the case of the Rosetta Stone, this measurement is proportional to the area lost in the rupture process because that part always increases (section \ref{entropy}). Future applications to other archaeological objects will be considered."

**Comment 7:** *There are obvious problems with the format of the chart. You need to carefully check the format of each icon and correct formatting errors in the chart.*

**Response:** We have, accordingly, changed the format of the chart in figures 4 and 6.

**Comment 8:** *There are some basic grammatical errors in the article.*

**Response:** Thank you for pointing this out. We went through the entire manuscript to eliminate grammatical errors.

**Comment 9:** *The original formula in the article is suggested to supplement the formula derivation process.*

**Response:** This comment of the referee was combined with comment 5. And that formula was better explained. Thank you for this suggestion.

In the same sense, we have given greater support to the rule of fractures related to the Griffiths criterion by incorporating explicit references to that theory.

**Comment 10:** *There are obvious errors in the structure of the article. It is recommended to recalibrate the structure of the article and indent the first line at the beginning of part 3.*

**Response:** We have adjusted the article's formatting to align with the PLOS ONE template. We took great care to meticulously review and restructure the entire article, paying close attention to the indentation at the commencement of each section. Your guidance has proven immensely helpful in enhancing the overall quality and presentation of the manuscript.

**Comment 11:** *At the end of the paper, the suggestions of future outlook are described, for example, the improvement ideas of the model and the process of data processing are described in detail.*

**Response:** Thank you for pointing this out. We have considered your comment, and it was incorporated with comment 6 in the conclusions section.

**Comment 12:** *The contribution description of the article is not obvious enough, so it is suggested to supplement the detailed contribution of the article.*

**Response:** Thank you for this suggestion. We have reinforced the contribution of the article in the conclusion section. Some applications of the model are described in the Introduction section (see the paragraphs Rolling stones in rivers, Crack networks, and Breakage of solid structures).
